# Successful prevention of organic solvent induced disorders: history  and lessons

**DOI:** 10.5271/sjweh.4155

**Published:** 2024-04-01

**Authors:** Maria Albin, Gunnar Johanson, Christer Hogstedt

**Affiliations:** 1 Institute of Environmental Medicine, Karolinska Institutet, SE-171 77 Stockholm, Sweden; 2 Division of Occupational and Environmental Medicine, Lund University, SE-223 63 Lund, Sweden

**Keywords:** chronic neurotoxicity, occupational exposure limit, painter, social partner, toxic encephalopathy, union advocacy

## Abstract

In this discussion paper, we describe the history of the science and societal action resulting in the mitigation of neurotoxic disorders from exposure to organic solvents at the workplaces in Sweden. When alkyd paints were introduced in large scale in construction painting in the 1960s and 1970s, Scandinavian unions voiced increasing concern as members reported symptoms like headache and vertigo, supported by participatory studies and case studies. Although acute and chronic neurotoxic effects were established for some specific solvents such as carbon disulphide, this was not the case for those used in the new paints. Union advocacy promoted formal epidemiological studies, providing increasing evidence for chronic neurotoxicity at levels far below current occupational exposure levels. The results were widely disseminated and accepted and led to concerted action with preventive measures, most importantly substitution of the organic solvents in paints for indoor use, but also drastic reductions in occupational exposure limits. The findings also resulted in funding of further research on solvent toxicity and the establishment of expert groups to advice authorities on occupational standards for exposure to chemicals. The substitution strategy was subsequently adopted in many other countries and occupational exposure limits were lowered, although several years or even decades later.

While the societal context in Sweden was unique in many ways, we conclude that there are lessons to be learned from this preventive success when addressing current challenges.

## Emerging concerns among painters

Construction painting post World War II saw rapid changes in many countries with the introduction of new paints. In Sweden, the rapidly drying and easily applied alkyd paints (binder, pigment, organic solvent), developed already around 1925 (1), became commonly used from the 1950s and were reportedly accompanied by instances of acute symptoms (headache, nausea, vertigo). In parallel, organization of painting work changed, in Sweden most markedly driven by the major effort in the 1960s and 1970s to eliminate the shortage of housing (building one million new dwellings in ten years) and provide needed facilities for the expanding welfare state (childcare, schools and hospitals). Changes included: larger painted surfaces, increase of spray painting, poorly ventilated spaces, and increased time pressure doing the job (2).

The early 1970s saw increasing concern among Swedish painters and their union regarding health risks associated with organic solvents in paints. The context was one of political upheaval in Sweden, as elsewhere, including several large strikes with demands for better working conditions (3). In 1960, a collaborative body (producers, employers, painters' union) introduced hazard labelling of the paints to support appropriate handling and protective equipment, but painters still reported symptoms, and concerns were raised with paint producers and occupational medicine experts. Replacement of solvent-based paints with safer products was one of the main demands coming out of the Swedish painters' union's national congress in 1974. The employer side additionally expressed a fear that the public debate on painters' hazards would weaken recruitment at a time of high demand (2).

Findings from Denmark influcenced the discussion. Medical students had published a first report on central nervous system (CNS) symptoms among 20 construction painter apprentices (4), followed by a questionnaire survey of 384 painters the next year (5, 6). In Sweden, the clinic of occupational medicine in Örebro reported an increasing number of painters with symptoms in the early 1970s (2).

## Emerging evidence of chronic health effects

Adverse effects of specific organic solvents have long been documented in the scientific literature. For example, already in 1856 Delpech reported acute mental confusion resulting from exposure to carbon disulphide. Chronic effects of solvents were accepted as a notifiable disease, eg, in the UK since 1924 (7).

Chronic CNS effects of organic solvents in general were also acknowledged in standard textbooks, eg, by Baader (8). These effects were attributed to the lipophilic properties of the solvents and included irritability, low mood, and anxiety, often summed up as 'nervousness', and occasionally also accompanied by symptoms from peripheral nerves. Efforts were made to disseminate this knowledge. For example, an informative leaflet from the Swedish National Institute of Public Health stated in 1942 that most organic solvents had a narcotic effect and could cause damage to the nervous system, which could be chronic and serious after prolonged exposure as they easily dissolved in its fatty tissue (9). The Swedish National Insurance Authority (Riksförsäkringsanstalten) awarded compensation to workers exposed to trichlorethylene in 18 cases from 1937–1941 indicating that the toxicity was known and acknowledged (10). In the 1960s, a series of severe acute intoxications (including loss of consciousness) occurring after glueing floor mats in confined spaces received much attention (10). However, the construction painters' reported acute symptoms that were milder and chronic adverse CNS effects were not documented among them at that time.

## New evidence in the 1970s and 1980s

Evidence on CNS effects was collected in a series of clinical, epidemiological, and toxicological studies, mainly performed in the Scandinavian countries from the 1970s onwards.

Cognitive impairment and neuropsychological symptoms among house painters were described in case series in Sweden (11) and Denmark (12), with cases categorized as psycho-organic syndrome and chronic toxic encephalopathy, respectively. The clinical observations were confirmed in cross-sectional studies in the Nordic countries among construction (1, 13–15) and car painters (16). An alarming finding was that health effects occurred well below the occupational exposure limits (OEL) at the time (16), triggering a debate on causality.

Epidemiological studies with a longitudinal approach found an increased risk for disability pension due to neuropsychiatric disorders among house painters (17, 18) and cabinetmakers using lacquers and varnishes (19).

While these early observations of health effects among painting trade workers were consistent, they remained controversial outside Scandinavia (20). The British Petroleum Group Occupational Health Centre's critical review carried out for the American Petroleum Institute (21) listed doubts based on lack of clinical, pathological, and neurophysiological findings, insufficient validation of questionnaires and psychometric tests for epidemiological studies, and concluded that the higher prevalence of health problems in painters may be due to other factors (ie, age, stress, environmental toxins, alcohol and medications) rather than to solvent exposure. Moreover, it was not clear if exposure in construction painting was associated with manifest dementia or mild cognitive impairment only (20). The World Health Organization (WHO) workshop in Copenhagen in 1985 (22) was an important step forward in developing a clinical classification of the findings, although it did not reach full consensus since one participant argued that the "painter syndrome" was only recognized in the Scandinavian countries and should first be internationally established. Long-term follow up of a national Swedish cohort of patients with chronic toxic encephalopathy confirmed that the cognitive impairment largely remained many years after cessation of exposure and affected both workability and everyday life but was not progressive. Importantly, among those with symptoms but no impairment in psychometric tests, symptoms were reduced or regressed if exposure ceased (23), highlighting the importance of early intervention. The original observations that symptoms and cognitive impairments may in milder cases be partly reversible, but remain chronic, albeit non-progressive, after heavier exposure, are now widely accepted (24).

**Figure 1:**
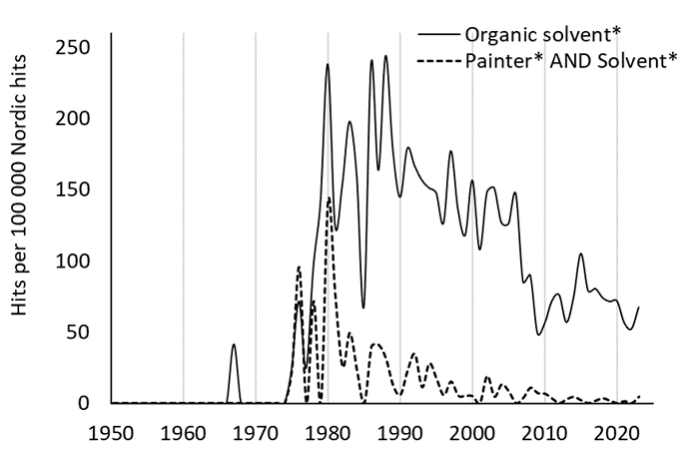
Nordic publications (hits) in PubMed (with authors from or dealing with the situation in Denmark, Finland, Norway or Sweden) addressing organic solvent(s) (___) and painter(s) and solvent(s) (- - -). The hits are expressed relative to the total number of Nordic hits. Constructed with PubMed by Year (https://esperr.github.io/pubmed-by-year).

**Figure 2:**
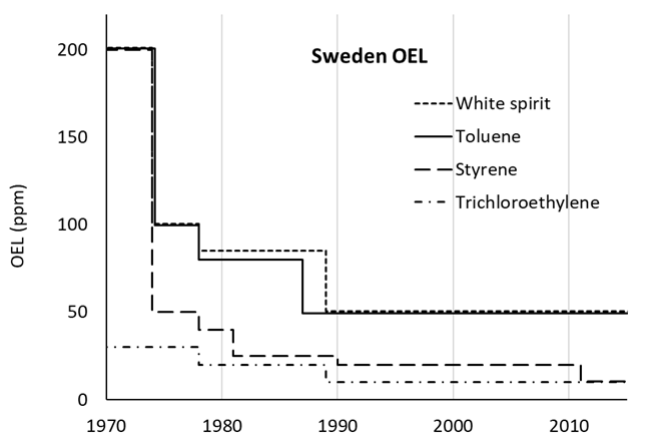
Development of occupational exposure limits (OEL) for selected organic solvents. Based on a review of instructions and provisions of the Swedish Work Environment Authority and its precursors (45–53). The OEL for white spirit 1970-1974 is the ACGIH TLV for Stoddard solvent at the time (54). The reduced OEL towards the end of the 1980s coincide with a Swedish Government initiative resulting in a reduction of the OEL of 25 organic solvents, among them trichloroethylene and white spirit (26).

The increased evidence of chronic health effects among painters elicited research on the health effects of a number of different solvents, not primarily related to paints or painters' exposure (figure 1). Also, other health-related aspects were investigated, such as uptake, organ distribution, biotransformation, excretion and biological exposure monitoring [see, eg, Åstrand's review (25)]. In addition, expert committees such as the Nordic Expert Group (initiated 1977 with funding from the Nordic Council of Ministers) and the Swedish Criteria Group were formed to supply the Nordic authorities with research-based evidence for setting OEL and other regulatory decisions. Commissioned by the government in 1987–1988, the Swedish Work Environment Authority investigated the possibility of reducing the OEL of organic solvents to one half (26, 27). Altogether, these actions led to a dramatic lowering of the OEL in the 1970s and 1980s (figure 2).

Worker unions in Sweden, especially the painters' union, were strong advocates of measures to reduce solvent exposure. Paint producers became increasingly aware of the need to provide products with a low solvent content, especially for indoor building painting (2). The urgency of the matter was evident also from the number of reported occupational diseases due to exposure to organic solvents: 250-400 per year between 1980 and 1984 (27).

The efforts were supported by dedicated research communication and funding: A joint program committee between the funding agency dedicated to work environment research (Arbetarskyddsfonden) and the Committee for occupational medicine within the Medical Research Council summarized the current evidence in 1979 (28). Based on this report, an ambitious campaign was launched, in collaboration with the social partners, on the health risks and technical possibilities to reduce exposure, basically following the hierarchy of controls (9). Also, funding was prioritized for development of water-based paints, lacquers and glues in the following three years. By 1984, <10% of the total paints used in construction were solvent-based (2). Finally, the painters' union stepped up their activities in 1986–1987 and achieved an agreement with employers stipulating that the use of solvent-based paint should be highly restricted (27). Interestingly, it also stipulated that all union members should be offered 16 hours of training on how to apply water-based wood paints, which partly required another technique. Of 16 000 union members, 10 000 received the training within a year, which facilitated the shift to water-based paints (2).

According to Hollander (2), the strategy to reduce solvent exposure in Denmark also saw a relatively early state intervention in 1982, with a ruling that given equal performance, the less dangerous paint must always be chosen based on a classification of hazard from inhalation and dermal contact.

Ultimately, the Swedish Government also took firm action. The use of solvent-based products for indoor construction painting was prohibited in 1987 (29) and the aforementioned investigation exploring the possibility to reduce the OEL of organic solvents was initiated the same year (26, 27). The investigation (27), together with the previous activities (research, expert committees) resulted in a progressive and substantial reduction of the OEL (figure 2). Meanwhile, Sweden was not alone. Although starting from higher values, similar reductions were seen around the same time in the American Conference of Governmental Industrial Hygienists' (ACGIH) threshold limit values (TLV) (30) and in Norway (26).

**Figure 3:**
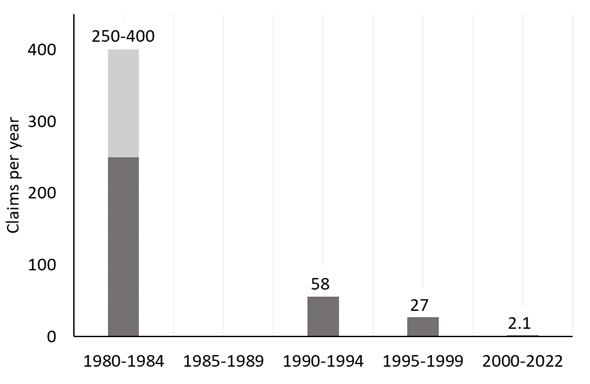
Number of solvent exposure injury claims in Sweden. Data for 1980–1984 are from a report from Arbetarskyddsstyrelsen in 1988 (36), which states that the claims varied between 250–400 per year, whereof 80% were approved. We were unable to find data for 1985–1989. Data for 1990–2022 show approved claims from the work injury register at AFA Försäkring (personal communication, Maria Schütt and Elin Henriksson, 31 October 2023).

The continuous reduction in exposure is also reflected in the number of approved cases of chronic toxic encephalopathy due to exposure to organic solvents, which were halved from the first to the last part of the 1990s and dramatically reduced after the millennium shift (figure 3). Approximately one third were construction painters over the entire period (Maria Schütt and Elin Henriksson, AFA Försäkring, personal communication, 31 October, 2023).

Recent follow-up studies confirm that the reduced exposure levels have influenced the health outcome. Järvholm & Burdorf (31) reported that the increased risk for disability pension due to neurological disorders among construction painters decreased from the early 1990s, compared to other construction workers. Similarly, the cases of chronic solvent encephalopathy in Finland dropped seven-fold, from 8.6 to 1.2 per million employed during 1995–2007 (32). Decreases were also seen in other European countries (33).

While much of the cognitive impairment associated with construction painting in Scandinavia and many other countries has been eliminated by substitution with less dangerous products (ie, water-based paints), CNS-effects from organic solvents in working life may still be an issue. Thus, a population-based study of life-time occupational exposures in a representative sample of the French national population indicates that cognitive effects from organic solvents still are a concern among both men and women (34)

## Discussion

### A unique setting

At the time when the organic solvent issue arose, Sweden was not yet part of the European Union and thus ruling and negotiating the work environment was done entirely in a national context. Structurally, this context was characterized by high unionization, and a strong bargaining power among construction painters. Also, members of the blue-collar unions were collectively affiliated to the Social Democratic Workers' party, which had been in government for decades and had a prominent voice in setting out its policies. The late 1960s and 1970s were, however, also characterized by discontent and large strikes over working conditions that were seen as increasingly stressful and inhumane, thus sensitizing the institutional framework to workers' demands and putting occupational safety and health on the public agenda. In this setting, a good work environment (in contrast to, eg, better salaries) was increasingly seen as a common, not inherently confrontational, cause for workers and employers, promoting health, wellbeing, productivity and as a strength in the competition for skilled workers. While both the institutional and political contexts were unique, we note the enormous potential in the transformative force of focused union struggle and close social partner and state agency dialogue in successfully solving a common work environment issue.

Preventive measures were unnecessarily delayed.

Initially, the observations of chronic CNS-effects at levels of exposure below the current OEL and for solvents without peripheral neurotoxicity were met with skepticism in Scandinavia, and the earlier acceptance of CNS-toxicity for the overall group of organic solvents was not brought forward. Outside Scandinavia, the lack of reported domestic cases of chronic toxic encephalopathy tended to be seen as indicating no or low risk – and the research findings were questioned as "Scandinavian disease" although they had been published in well-established international peer-reviewed journals. We recognize these reactions as the fallacy of "no data – no problem – no action" and "merchants of doubt" (35). For example, the OEL for various solvents were drastically reduced in Sweden in the 1970s and 1980s (figure 2) whereas in the UK, the regulatory authorities waited until the 1990s before reducing the OEL, in parallel with most of the EU member states (36).

### Facilitating substitution

Hazard reduction strategies relied mainly on personal protective equipment up to the second half of the 1970s, but with little effect on the number of reported cases with chronic CNS-effects. Substitution of the dangerous components proved to be the solution. Ingeniously, the shift was facilitated by offering the workers free training in the use of the new water-based products, thus avoiding loss of earnings and quality problems. This may still serve as a model for smooth transitions to less harmful products and techniques.

### Applicability to current challenges

Measures to reduce exposure to highly toxic substances have historically relied too long (eg, asbestos, pesticides) or continues to rely (eg, crystalline silica) on personal protective equipment. Meanwhile, other control measures have largely been neglected.

The legendary occupational toxicologist Alice Hamilton (37) described the resistance in the US to accept other countries' findings on carbon disulphide toxicity in the rayon viscose industry: "There are few industrial diseases which move one's sympathy more than does carbon disulphide poisoning. … As always, I found it impossible to believe that an industry which in the countries of its origin was looked on as a dangerous trade was perfectly harmless over here, since we were using the same processes as the Europeans and the Japanese. Again and again I had heard that claim made in connection with some industrial poison but always it proved to be quite unfounded."

Lack of domestic evidence of cases are to a surprising extent still a barrier to prevention. A current and striking example is kitchen tops made of artificial stone where epidemics of severely disabling and progressive silicosis with rapid onset among young workers have been reported from a series of countries world-wide (38). Australia will ban the product due to the inherent highly toxic properties (39), California has issued an emergency temporary standard (40) and The Netherlands have submitted an initiative to ECHA for restriction and enforced regulation (38). Meanwhile, acceptance of the evidence and measures to control exposure remain absent in most European countries, including Sweden.

Today, the Scandinavian societies/countries seem less receptive than 50 years ago to advocacy for worker safety and health due to lower unionization and weaker worker bargaining power, accompanied by reduced governmental resources for occupational toxicology and hygiene (41, 42). This is worrying, given the rapid transformation to a circular economy and transition to new energy supply systems, which may result in new or increased occupational hazards (eg, critical minerals and metals such as lithium, cobalt, nickel and manganese (43). On the other hand, tools to identify hazardous exposures have developed enormously, including eg, non-invasive self-administered exposure monitoring, and biomarkers of exposure and early effect. This will hopefully permit more rapid identification and assessment of the new hazards and, in the end, more rapid risk communication and management. Independent expertise and resources need to be at hand to use these tools and the risk assessment thus putting a sustainable funding for maintaining this independent competence on the agenda.

### Summary and lessons learned

Our brief historical exposé aims to try to identify the major factors behind the success with solvent exposure mitigation and prevention of neurotoxic disorders in Sweden. The short descriptions below can also be regarded as lessons we learned. Many of them have been components in other work environment success stories, although the context is different at varying times and different countries.

Case studies were informative by pointing to possible associations between exposure to organic solvents and chronic neurotoxic effects and formed the basis for hypothesis.
Reports initiated by trade unionists and performed by medical students similar to the science shop movement (44) were influential and motivated larger studies.
The pressure from the painters' and other trade unions to lower and ban solvent exposure was strong and effective and resulted in actions from the legislative authorities including OEL setting.
Research programs were generously funded and included exposure assessment, longitudinal health studies, toxicological studies, and preventive measures.
Research reports with conclusive results were not only published in international scientific journals but also made available in Swedish to governmental authorities, employers, trade unions and concerned communities. This stimulated the creation of action oriented, cooperative committees between the social partners, government agencies and researchers.
